# Evaluating Pulmonary Arterial Pressure in Angus Yearling Bulls at High Elevation: Associations with Birth Traits and Growth Performance

**DOI:** 10.3390/ani15091302

**Published:** 2025-04-30

**Authors:** Kaylen Stearns, Hannah DelCurto-Wyffels, Sam Wyffels, Megan Van Emon, Noah G. Davis, Taylre Sitz, Tim DelCurto

**Affiliations:** 1 Department of Animal and Range Sciences, Montana State University, Bozeman, MT 59717, USA; hannah.delcurto@montana.edu (H.D.-W.); sam.wyffels@montana.edu (S.W.); megan.vanemon@montana.edu (M.V.E.); noahdavis3@montana.edu (N.G.D.); 2 Sitz Angus Ranch, Harrison, MT 59735, USA; taylreesitz@gmail.com

**Keywords:** beef cattle, heart−lung function, performance

## Abstract

The objectives of this study were to model the relationship between pulmonary arterial pressure (PAP) estimates and birth weight, gestation length, and growth traits of developing bulls. Pulmonary arterial pressure estimates were collected from Angus yearling bulls ranging in age from 12 to 18 months from a Montana-based Angus operation (>1600 m elevation, 5406 yearlings/18-month-old bulls from 2016–2023). Pulmonary arterial pressure was positively correlated with birth weight (*p* < 0.01) and gestation length (*p* < 0.01). No relationship was observed between bull PAP and weaning weight (*p* = 0.26). Pulmonary arterial pressure scores were negatively correlated with yearling weight (*p* = 0.04). Additionally, PAP estimates were negatively correlated with birth-to-weaning gains (*p* < 0.01) and weaning-to-yearling gains (*p* = 0.02). Lastly, PAP scores were negatively correlated with birth to yearling growth (*p* < 0.01). Sire lines influenced offspring PAP scores (*p* < 0.01), and dam−sire lines tended to have an effect on offspring PAP scores (*p* = 0.09). In summary, PAP scores were related to birth weight, gestation length, yearling weight, and growth from birth to yearling age. Our findings suggest that selection for higher growth should also emphasize selecting for lower PAP scores.

## Simple Summary

As the cattle sector puts more emphasis and focus on heart−lung function, it is important to understand the relationship between pulmonary arterial pressure (PAP) scores and performance measurements. This study characterized the relationship between PAP scores and gestation length, birth weight, and growth traits. The results showed that as gestation length and birth weight increased, PAP scores also increased. In comparison, bulls with increased yearling weights and birth to yearling gains had decreased PAP scores. No relationship was observed between PAP scores and weaning weight. By evaluating these relationships, beef cattle producers can gain better insights and tools for selecting cattle for high-elevation production systems.

## 1. Introduction

Pulmonary arterial pressure (PAP) determines an animal’s susceptibility to High Altitude Disease (HAD), also known as Brisket Disease, High Mountain Disease (HMD), and right-sided heart failure (RHF) [1]. It is a non-infectious disease that causes pulmonary vasoconstriction due to hypoxia [2]. High Altitude Disease impacts beef cattle production at elevations above 1500 m due to decreased oxygen saturation in the atmosphere [1]. Clinical signs of HAD include brisket edema, lethargy, jugular vein distention, diarrhea, poor appetite, and death [3]. Currently, no treatment or cure exists for HAD [3]. The only recommendation is to move the affected animals to lower elevations with more tolerable oxygen conditions [3]. In 2012, it was estimated that 1.5 million cattle resided in high-elevation production systems [4]. High Altitude Disease accounts for 3–5% of the annual calf death loss at altitude [1] and results in an economic loss of approximately $60 million annually [4].

Bovids have an increased susceptibility to HAD because of their pulmonary system [1,5]. Pulmonary vascular shunting is a mechanism that moves pulmonary blood flow away from poorly oxygenated tissues in the lung to areas of higher oxygen concentration [5]. Shunting occurs in all animals during hypoxia [1] and causes persistent exposure of the pulmonary vasculature to increased pressure [6]. This constant increase in exposure and pressure leads to remodeling of the pulmonary arteries caused by hypertrophy and muscularization [5,7]. Eventually, this leads to loss of function within the peripheral pulmonary arteries and dilation of the right ventricle, followed by heart failure [1,5,7]. The bovine pulmonary system experiences the greatest amount of pulmonary vascular shunting across the tested species of llamas, dogs, and cats [6]. In addition, the bovine lung is relatively small compared to its body weight and has a smaller lobulated anatomical pattern, which increases the likelihood of cattle suffering from severe pulmonary hypertension and loss of function [8].

Seedstock producers use PAP scores as a tool to market their cattle to producers who operate at high elevations or those who use heart−lung function as a selection criterion [9]. Additionally, selection for growth and performance has continually trended upward over the past four decades in Angus cattle [10]. Consequently, the cattle sector is placing more emphasis on heart−lung function as RHF continues to increase at moderate elevations, which comes at a time when carcass weights have drastically increased [11,12,13].

There is minimal research on the relationship between PAP measurements and cattle performance, and previous research has yielded conflicting results. The objectives of this study were to model the relationship between PAP and gestation length, birth weight, and growth traits of developing Angus bulls. In addition, this study compares mean PAP scores of the most common sire and grandsire lines from progeny within the data set. Increased knowledge of PAP scores and the relationship between performance traits and PAP estimates can allow for a better understanding of the selection goals in beef cattle.

## 2. Materials and Methods

We collaborated with a Montana-based Angus operation to gather data on 5406 heads of 12-to 18-month-old purebred Angus bulls sold in their annual production sales from 2016 to 2023. Bulls were raised and resided at elevations greater than 1600 m. Notably, this operation has two separate locations that function as two separate ranches. Bulls from Operation A are sold in the December production sale (Fall sale), and PAP tested at 18 months of age. Bulls are weaned in a pasture setting in the fall and then sent to the ranch feedlot for the winter. In spring, bulls are turned out on forest allotments (≈2590 m elevation) to graze. They return to the feedlot in the fall, where they are PAP tested following their return from elevation. While at the feedlot, they receive a light growing ration until the bull sale. Bulls from Operation B are sold in the March production sale (Spring sale), and PAP tested at 12 months of age. Bulls from Operation B are weaned into a feedlot (≈1600 m elevation) in early September. The bulls are sorted into groups by weight and started on a high-roughage diet. From there, concentrates of corn and dried distillers grains are gradually added to the ration. Bulls are PAP tested in the middle of December. Through a right-heart catheterization procedure, [14], a PAP test measures the resistance of blood flow through the lungs [15]. Measurement of PAP has been routinely performed by a licensed veterinarian at beef cattle operations at high elevations since the 1980s. In 2019, the American Angus Association (AAA) established a PAP expected progeny difference (EPD). Therefore, an AACUC was not obtained for this study. Similar to human blood pressure, PAP scores are measured in millimeters of mercury (mmHg) [1]. Scores range from 30 mmHg to greater than 50 mmHg, with lower scores being more desirable (30–40 mmHg) [1]. Pulmonary arterial pressure scores are influenced by several factors, including age, sex, genetics, and environmental conditions [1,16,17,18,19].

The data gathered included individual bull identification number, sale, sale year, birthdate, birth weight, weaning weight, yearling weight, PAP score, sire’s AAA registration number, dam’s AAA registration number, and progeny’s AAA registration number. For a subset of the data, 485 bulls from two years of fall sale, artificial insemination dates were obtained. Gestation length was calculated as the difference between the birthdate and the artificial insemination date. DNA tests were used to confirm the parentage of the calf for all bulls.

Phenotypic correlations were used to determine the relationship between PAP estimates and gestation length, birth weight, and growth traits. It was hypothesized that the relationship between PAP and performance traits could be linear, asymptotic, or quadratic in nature [20]. Candidate models were developed to reflect the nature of each response variable to PAP using generalized linear mixed models with a fixed effect of the variable of interest as either linear, asymptotic, or quadratic in nature and year as a random intercept. Akaike’s Information Criterion adjusted for small sample sizes (AICc) was used to evaluate the support for competing models (Table 1; “AICcmodavg” package for R) [21]. Once the nature of the relationship was determined for each variable of interest, candidate model sets using generalized linear mixed models were developed, reflecting multiple hypotheses on the relationship between each of the performance metrics, gestation length, and birth weight, including the fall versus spring sale and its associated interaction with each of the variables of interest on bull PAP scores using the packages “lme4” and “lmerTest” (packages for R) [22,23]. The year was treated as a random intercept. Akaike’s Information Criterion adjusted for small sample sizes (AICc) was used to evaluate support for competing models reflecting hypotheses about the effects of sale (age at the time of testing) and performance traits (Table 2; “AICcmodavg” package for R) [21]. We excluded models with ΔAICc ≤ 2 that differed from the top model by a single parameter if the confidence intervals of the parameter estimates overlapped 0 (i.e., were noninformative) [24]. Model-averaged estimates of beta coefficients were used when multiple models were supported (“MuMin” package for R) [25]. Model fit was evaluated using marginal and conditional r^2^ [26]. All models and relationships were evaluated using R version 1.7.2 [27].

All sires and maternal grandsires with a minimum of 100 offspring (15) in our data were identified and used to characterize the effect of the sire line on PAP scores. Progeny PAP data were analyzed using generalized linear mixed models (“lme4” and “lmerTest” packages for R) [22,23] in an ANOVA framework (“car” package for R) [28], including sire line as a fixed effect and year as a random intercept. All data were plotted and natural log-transformed, if needed, to satisfy the assumptions of normality and homogeneity of variance. Significance was determined at an alpha of <0.05, with a tendency discussed at an alpha of >0.05 and <0.10. The Tukey method was used to separate means when alpha was <0.05 (“emmeans” package for R) [29]. All statistical analyses were performed using R [27].

## 3. Results

There were no significant relationships (*p* = 0.26) between PAP and weaning weight.

All other variables displayed asymptotic relationships with PAP (Table 1).

**Figure 1:**
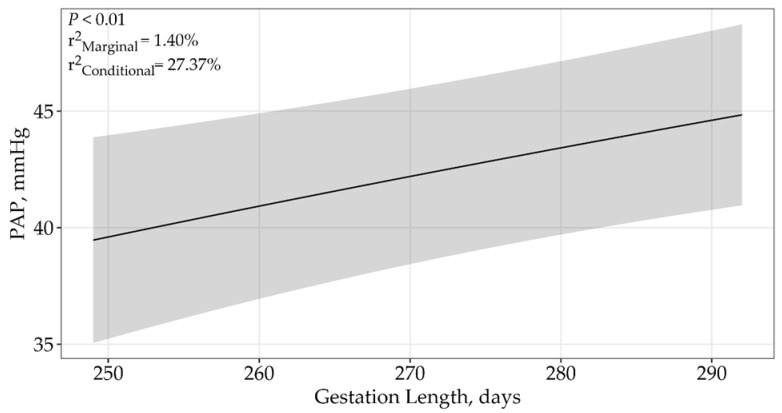
Predicted relationship (±95% CI represented in the shaded area) between PAP scores and gestation lengths for 485 eighteen-month-old bulls raised and tested above 1600 m in southwestern Montana, USA. This relationship was best described using an asymptotic model (*p* < 0.01; β = 33.74 ± 11.25). The solid line represents the relationship between PAP scores and gestation length for the Fall sale hosted by Operation A.

Gestation length displayed an asymptotic relationship (*p* < 0.01) with PAP, and a single top model received 100% support among candidate models when determining the effects of gestation length on PAP (Table 2). A model containing only gestation length (β = 33.74 ± 11.25; Figure 1) showed that PAP increased with increasing gestation length. The top model containing all supported variables had a conditional r^2^ of 27.37% and a marginal r^2^ of 1.40%, suggesting that gestation length accounts for 1.40% of the variation in PAP scores. However, it should be noted that gestation lengths were only collected for two Fall sales.

**Figure 2:**
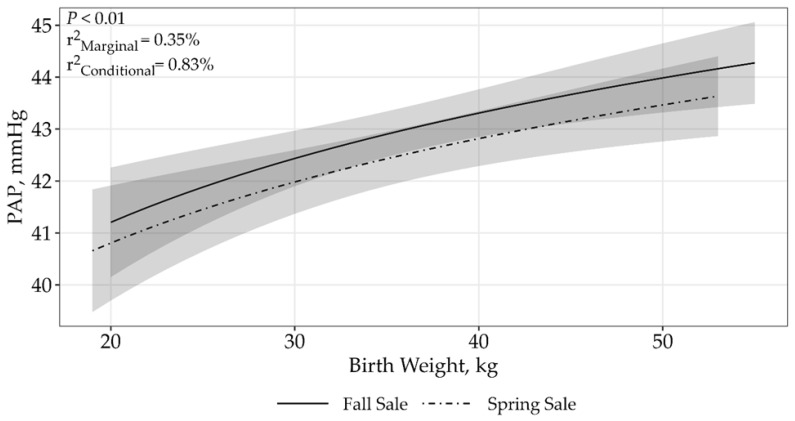
Predicted relationship (±95% CI represented in the shaded areas) between PAP scores and birth weight for 5406 twelve to eighteen-month-old bulls raised and tested above 1600 m in southwestern Montana, USA. The relationship was best described by an asymptotic model (*p* > 0.01; β_Fall Sale_ = 3.04 ± 0.82, β_Spring Sale_ = 2.91 ± 0.81). The solid line represents the Fall sale hosted by Operation A, while the dashed line represents the Spring sale hosted by Operation B.

Models containing birth weight and sale accounted for 100% of the support among the candidate models (Table 2). Pulmonary arterial pressure was asymptotically related to birth weight by sale interaction (*p* > 0.01; β_Fall Sale_ = 3.04 ± 0.82, β_Spring Sale_ = 2.91 ± 0.81; Figure 2), where PAP increased with increasing birth weight across both sales. The top model containing all supported variables had a conditional r^2^ of 0.83% and a marginal r^2^ of 0.35%, suggesting that birth weight and sale only accounted for 0.35% of the variation associated with PAP.

A single top model containing weaning weight received 86% of the relative support of data when determining the effects of weaning weight on PAP (β = −0.96 ± 0.86; *p* = 0.26); however, this model may be noninformative as the confidence intervals of the effect overlap 0.

**Figure 3:**
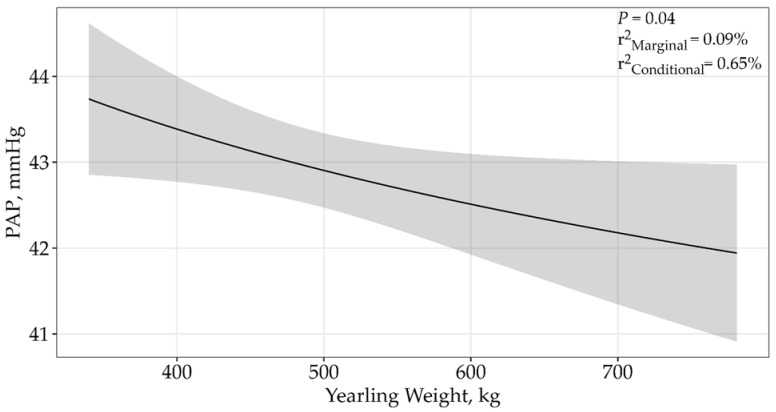
Predicted relationship (±95% CI represented in the shaded areas) between PAP scores and yearling weight for 5406 twelve to eighteen-month-old bulls raised and tested above 1600 m in southwestern Montana, USA. This relationship was best described by an asymptotic model (*p* = 0.04; β = −2.16 ± 1.05). The solid line represents the relationship between PAP scores and yearling weight. There was no difference between sales.

A single top model containing yearling weight received 93% support among the candidate models (Table 2; *p* = 0.04; β = −2.16 ± 1.05; Figure 3), where PAP decreased as yearling weight increased. The top model containing all the supported variables had a conditional r^2^ of 0.65% and a marginal r^2^ of 0.09%. Therefore, the yearling weight accounted for only 0.09% of the variation associated with PAP.

**Figure 4:**
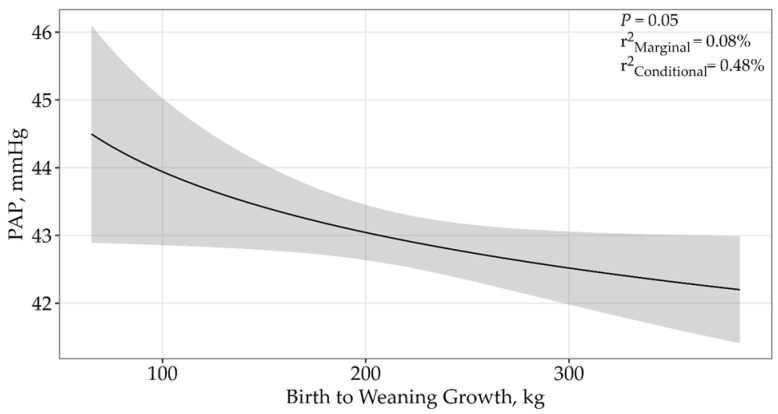
Predicted relationship (±95% CI represented in the shaded areas) between PAP scores and growth from birth to weaning for 5406 twelve to eighteen-month-old bulls raised and tested above 1600 m in southwestern Montana, USA. This relationship was best described by an asymptotic model (*p* = 0.05; β = −1.29 ± 0.65). The solid line represents the relationship between birth to weaning growth and PAP scores. There was no difference between sales.

Birth to weaning growth displayed an asymptotic relationship (*p* = 0.05), and the single top model received 91% of the relative support of data when determining the effects of birth-to-weaning gains on PAP (Table 2; β = −1.29 ± 0.65; Figure 4), where PAP decreased with increasing birth to weaning weight growth. The top model containing all supported variables had a conditional r^2^ of 0.48% and a marginal r^2^ of 0.08%.

**Figure 5:**
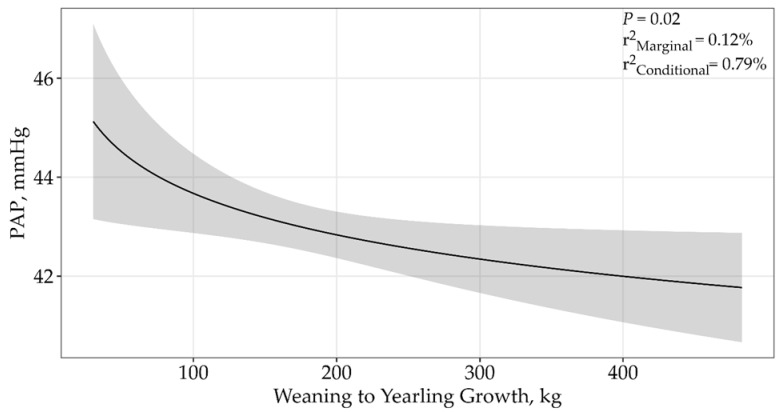
Predicted relationship (±95% CI represented in the shaded areas) between PAP scores and weaning to yearling growth for 5406 twelve to eighteen-month-old bulls raised and tested above 1600 m in southwestern Montana, USA. This relationship was best described by an asymptotic model (*p* = 0.02; β = −1.21 ± 0.54). The solid line represents the relationship between PAP scores and weaning to yearling growth. There was no difference between sales.

A model containing weaning to yearling growth received 93% support among candidate models (Table 2; *p* = 0.02; β = −1.21 ± 0.54; Figure 5), where PAP decreased as weaning-to-yearling growth increased. The top model had a conditional r^2^ of 0.79% and a marginal r^2^ of 0.12%, suggesting that weaning to yearling growth only accounted for 0.12% of the variation associated with PAP.

**Figure 6:**
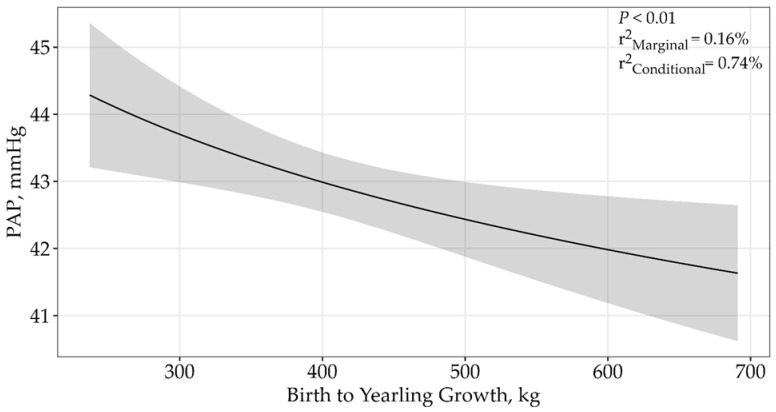
Predicted relationship (±95% CI represented in the shaded areas) between PAP scores and weaning to yearling growth for 5406 twelve to eighteen-month-old bulls raised and tested above 1600 m in southwestern Montana, USA. This relationship was best described by an asymptotic model (*p* < 0.01; β = −2.48 ± 0.90). The solid line represents the relationship between PAP scores and birth to yearling growth. There was no difference between sales.

An asymptotic relationship was displayed by a single top model containing birth to yearling weight (*p* < 0.01) and received 96% of the relative support of data when determining the effects of birth-to-yearling growth on PAP (Table 2). A model containing only birth-to-yearling gains (β = −2.48 ± 0.90; Figure 6) was supported, in which PAP decreased with increasing birth-to-yearling growth. The top model containing all supported variables had a conditional r^2^ of 0.74% and a marginal r^2^ of 0.16%. Based on our study, this suggests birth to yearling weight only accounts for 0.16% of the variation associated with PAP.

### 3.1. Sire Line Progeny Analysis

**Figure 7:**
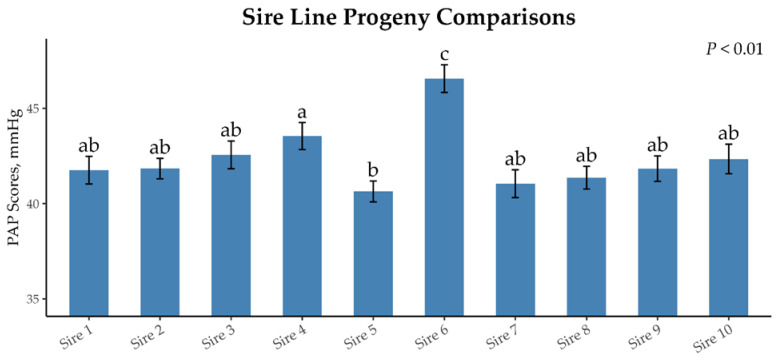
Mean PAP scores of sires with >100 progeny within the dataset. Sires that do not share a common letter differ (*p* < 0.01).

We identified 10 sires with at least 100 offspring, for a total of 1532 offspring. The mean PAP scores, range of PAP scores, and coefficient of variation for each sire are presented in Table 3. The range between the progeny of the sire line with the greatest PAP score and that of the least was 5.92 mmHg. The mean PAP scores of the offspring differed by sire line (*p* < 0.01; Table 3; Figure 7). Offspring from sire 6 had the greatest mean PAP (46.56 mm Hg), which was greater than that of offspring from the eight other sires (*p* < 0.01). Offspring from sire 5 had the lowest mean PAP (40.64 mm Hg), but it was only lower than that of offspring from sires 4 and 6 (*p* < 0.01). The actual sire EPDs and coefficient of variation of PAP scores are listed in Table 3 for reference.

### 3.2. DamSire Line Progeny Analysis

**Figure 8:**
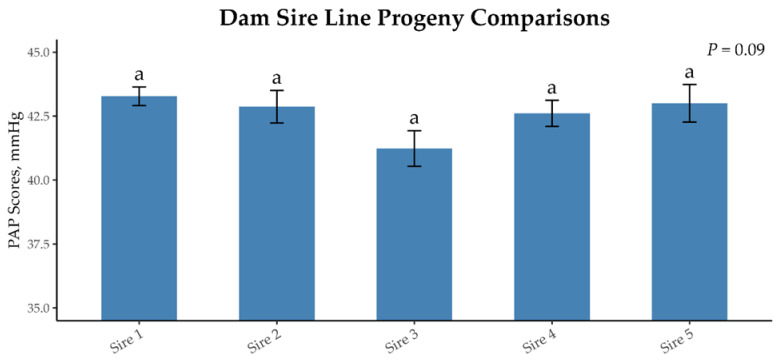
Mean PAP scores of dam sires (progeny’s maternal grandsire) with >100 progeny in the dataset. The mean PAP scores of progeny tended to be different (*p* = 0.09), but the post-hoc test did not detect any differences between the sires. Sires that do not share a common differ (*p* = 0.05).

Five dam sires with at least 100 grand progeny for a total of 1135 offspring (Table 4) were identified. The mean PAP score, range of PAP scores, and coefficient of variation for each sire were calculated. The range between the grand progeny of the sire line with the greatest PAP score and that of the least was 2.11 mmHg. There tended to be a difference in the average mean offspring PAP score (*p* = 0.09; Table 4; Figure 8), as sire 3 reported the lowest mean PAP scores in his grand progeny, while sire 1 reported the greatest mean PAP scores. The actual damsire EPDs and coefficient of variation of PAP scores are listed in Table 4 for reference.

## 4. Discussion

The use of performance driven Angus seedstock cattle to define the relationship between PAP scores and gestation length, birth weight, and performance traits provided a research model that uses real-world applications. The candidate models showed that there was a relationship between PAP and gestation length, birth weight, and yearling weight. Additionally, candidate models suggested relationships between birth-to-weaning growth, weaning-to-yearling gains, and birth-to-yearling growth with PAP. First, examining the relationship between PAP scores and gestation length revealed that as the length of gestation increases, PAP estimates also increase. It was hypothesized that shortened gestation lengths may increase PAP scores because previous research suggests that shortened gestation lengths can decrease lung maturity, which in turn may put a calf at greater risk for respiratory distress [30,31]. Our findings contradict those of a previous study by Foxworthy et al. [32], which showed that gestation length is a weak negative predictor of PAP scores. In our study, gestation length only explained 1.40% of the variation in PAP measurements.

The relationship between PAP scores and birth weight showed that as birth weight increased, PAP estimates increased. Two previous studies conducted by Colorado State University investigated the same relationship. In 2008, Shirley et al. [15] reported that PAP scores were moderately and positively genetically correlated with birth weight. Shirley et al. [15] concluded that selection for growth and performance may increase PAP scores. In 2016, Crawford et al. [33] reported a weak positive genetic correlation between PAP scores and birth weight. Based on their study, Crawford et al. [33] suggested that selecting for lower PAP scores may decrease birth weights. The results of these studies closely resemble those of our study. Our study indicated that birth weight accounted for 0.25% of the variation in PAP measurements.

The relationship between PAP scores and yearling weight suggests that as performance and gains increase, PAP estimates decrease. In addition, our analysis showed that bulls with increased gains from birth to weaning, weaning to yearling, and birth to yearling showed decreased PAP scores. Based on the inverse relationship found in our study, we concluded that bulls with improved heart -lung function will experience increased gains. From a biological standpoint, bulls with improved heart−lung function are better set to achieve greater performance than those with decreased heart−lung function. Previous work has yielded conflicting results. In a study conducted by Schimmel et al. [34] in 1981, there was a strong, negative genetic relationship (−0.75 ± 0.65) between PAP scores and yearling weights. In contrast, Crawford et al. [33] found a weak positive genetic relationship (0.12 ± 0.08) for yearling weight. A more recent study by Pauling et al. [10] suggested a low genetic correlation between yearling weight and PAP (0.01 ± 0.11). In our study, yearling weight only explained 0.09% of the variation in PAP measurements. Previous genetic correlations of post-weaning gains have indicated similar results (−0.10 ± 0.10) [33] and (−0.11 ± 0.12) [10]. Our phenotypic correlations suggested similar findings, as bulls with increased gains had lower PAP scores. The growth from birth to weaning explained 0.08% of the variation in PAP estimates, and the growth from weaning to yearling explained 0.12% of the variation in PAP scores. Additionally, growth from birth to yearling explained 0.16% of the variation in PAP measurements.

The characterization of mean PAP scores of progeny from sire lines and maternal grandsire lines with more than 100 offspring within the dataset showed that there is a relationship between a sire and an offspring’s PAP score, reinforcing the trait’s heritability. Pulmonary arterial pressure heritability estimates ranged from 0.20 to 0.46 [15,33,35,36]. Differences were observed across sire lines among the mean PAP scores of the progeny. The coefficient of variation (CV) provides predictability of that sire’s progeny’s PAP scores. Sires with a smaller CV for PAP scores generally produce offspring with less variability.

The findings of this study could help enhance beef cattle production in high-elevation systems. By better understanding how PAP scores are related to performance, beef cattle producers can make conscious bull selection decisions to achieve production goals based on their operations. Sire progeny showed consistency and little variability in PAP scores. This illustrates that sire selection for PAP makes a difference and has an impact on an operation.

## 5. Conclusions

As HAD continues to be a threat in the beef sector, it is important to understand the relationship between economically relevant traits and PAP scores. This research provides insight into how these relationships can impact beef production in high-elevation systems. This research showed a significant relationship between PAP and gestation length, birth weight, yearling weight, and weaning to yearling gain. However, these relationships explain very little variation in PAP estimates. The sire line summary demonstrates that sire selection does impact heart−lung function in offspring. Our results suggest that there is an effect between the sire and offspring PAP scores. Based on these findings, it is crucial to continue placing emphasis on heart−lung function in all production systems, thus allowing cattle to thrive in varied production environments. Our results suggest that selection for higher growth should also emphasize the selection of lower PAP scores. Therefore, additional research on beef cattle growth, performance, and heart−lung function is warranted.

## Figures and Tables

**Table 1:** Model selection for evaluating the nature (linear, asymptotic, or quadratic) of the relationship between PAP and performance metrics, gestation length, and birth weight in 12 to 18-month-old Angus bulls.

Model ^1^	K ^2^	AICc ^3^	ΔAICc ^4^	W_i_^5^	*p*-Value
Gestation Length					
ln(Gestation Length)	4	2783.65	0	1	<0.01
Gestation Length	4	2784.86	11.20	0	<0.01
(Gestation Length) ^2^	5	2806.20	22.55	0	0.85
Birth Weight					
ln(Birth Weight)	4	33,627.32	0	0.97	<0.01
Birth Weight	4	33,363.42	7.29	0.03	<0.01
(Birth Weight) ^2^	5	33,374.95	18.18	0	0.82
Weaning Weight					
ln(Weaning Weight)	4	33,627.32	0	1	0.24
Weaning Weight	4	33,638.66	11.35	0	0.26
(Weaning Weight) ^2^	5	33,658.47	31.15	0	0.72
Yearling Weight					
ln(Yearling Weight)	4	33,612.76	0	1	0.04
Yearling Weight	4	33,625.17	12.58	0	0.04
(Yearling Weight) ^2^	5	33,646.23	33.47	0	0.64
Growth from Birth to Weaning					
ln(Growth from Birth to Weaning)	4	33,215.41	0	0.99	0.03
Growth from Birth to Weaning	4	33,225.35	9.94	0.01	0.05
(Growth from Birth to Weaning) ^2^	5	33,243.76	28.35	0	0.23
Growth from Weaning to Yearling					
ln(Growth from Weaning to Yearling)	4	33,413.33	0	1	0.06
Growth from Weaning to Yearling	4	33,425.17	11.84	0	0.02
(Growth from Weaning to Yearling) ^2^	5	33,646.26	33.47	0	0.07
Growth from Birth to Yearling					
ln(Growth from Birth to Yearling)	4	33,131.04	0	1	<0.01
Growth from Birth to Yearling	4	33,143.26	12.21	0	<0.01
(Growth from Birth to Yearling) ^2^	5	33,164.16	33.12	0	0.81

^1^ Year is used as a random variable in all models. ^2^ K is the number of parameters. ^3^ Akaike’s Information Criterion adjusted for small sample size. ^4^ Difference in Akaike’s Information Criterion adjusted for small sample size compared to the best model. ^5^ Akaike weight.

**Table 2:** Model selection for evaluating the effects of performance metrics, gestation length, birth weight, fall versus spring sale, and associated interactions on 12 to 18-month-old purebred Angus bull PAP scores.

Model ^1^	K ^2^	AICc ^3^	ΔAICc ^4^	W_i_^5^	r^2^ m ^6^	r^2^ c ^7^	*p*-Value
Gestation Length							
ln(Gestation Length)	4	2783.85	0	1	1.40%	27.37%	<0.01
null	3	33,858.87	31,075.22	0	0%	0.50%	
Birth Weight							
ln(Birth Weight)	4	33,356.14	0	0.66	0.25%	0.77%	<0.01
ln(Birth Weight) × sale	5	33,357.46	1.33	0.34	0.35%	0.83%	<0.01
null	3	33,858.87	502.74	0	0%	0.50%	
Weaning Weight							
ln(Weaning Weight)	4	33,627.32	0	0.86	0.03%	0.47%	0.26
ln(Weaning Weight) × sale	5	33,630.91	3.59	0.14	0.09%	0.52%	0.67
null	3	33,858.87	246.11	0	0%	0.50%	
Yearling Weight							
ln(Yearling Weight)	4	33,612.76	0	0.93	0.09%	0.65%	0.04
ln(Yearling Weight) × sale	5	33,618.03	5.27	0.07	0.11%	0.62%	0.24
null	3	33,858.87	246.11	0	0%	0.50%	
Growth from Birth to Weaning							
ln(Growth from Birth to Weaning)	4	33,215.41	0	0.91	0.08%	0.48%	0.05
ln(Growth from Birth to Weaning) × sale	5	33,220.15	4.74	0.09	0.12%	0.51%	0.15
null	3	33,858.87	643.46	0	0%	0.50%	
Growth from Weaning to Yearling							
ln(Growth from Weaning to Yearling)	4	33,413.33	0	0.93	0.12%	0.79%	0.02
ln(Growth from Weaning to Yearling) × sale	5	33,418.36	5.03	0.07	0.13%	0.72%	0.07
null	3	33,858.87	445.55	0	0%	0.50%	
Birth to Yearling Growth							
ln(Birth to Yearling Growth)	4	33,131.04	0	0.96	0.16%	0.74%	<0.01
ln(Birth to Yearling Growth) × sale	5	33,137.19	6.15	0.04	0.16%	0.71%	0.04
null	3	33,858.87	727.83	0	0%	0.50%	

^1^ Year is used as a random variable in all models. ^2^ K is the number of parameters. ^3^ Akaike’s Information Criterion adjusted for small sample size. ^4^ Difference in Akaike’s Information Criterion adjusted for small sample size compared to the best model. ^5^ Akaike weight. ^6^ Marginal r^2^. ^7^ Conditional r^2.^

**Table 3:** Influence of 10 registered Angus bull sire lines on mean progeny PAP scores, the range of PAP scores, and the coefficient of variation for 1532 twelve to eighteen-month-old bulls raised and tested above 1600 m in southwestern Montana, USA.

Sires												
	1	2	3	4	5	6	7	8	9	10	SE	*p*-Value
**Number of offspring**	125	217	110	124	238	109	112	256	139	102		
**Average offspring PAP score**	41.57 ^ab^	41.84 ^ab^	42.56 ^ab^	43.55 ^a^	40.64 ^b^	46.56 ^c^	41.05 ^ab^	41.36 ^ab^	41.84 ^ab^	42.34 ^ab^	0.674	<0.01
**CV of offspring PAP score ^1^**	11.50	19.55	17.67	18.03	13.00	18.52	15.07	9.93	14.87	8.38		
**Sire PAP EPD ^2^**	−0.62	−1.10	+2.85	+0.41	−2.29	+2.22	−0.71	−1.94	−1.34	−0.51		
**EPD accuracy**	0.56	0.70	0.67	0.45	0.67	0.36	0.59	0.75	0.59	0.63		

^1^ coefficient of variation for PAP score, expressed as a percentage of the mean. ^2^ expressed in millimeters of mercury (mmHg), with a lower EPD being more favorable. ^a–c^ Means within a row that do not share a common superscript differ (*p* < 0.01).

**Table 4:** Influence of five registered Angus damsire lines on mean grand progeny PAP scores, the range of PAP scores, and the coefficient of variation for 1135 twelve to eighteen-month-old bulls raised and tested above 1600 m in southwestern Montana, USA.

Dam Sires							
	1	2	3	4	5	SE	*p*-Value
**Number of offspring**	523	144	115	246	107		
**Average offspring PAP score**	43.30	42.84	41.19	42.59	42.94	0.814	0.09
**CV of offspring PAP score ^1^**	17.88	18.15	13.47	15.47	16.31		
**Sire PAP EPD ^2^**	+1.96	+1.99	−0.29	−0.77	+3.38		
**EPD accuracy**	0.78	0.64	0.51	0.59	0.50		

^1^ coefficient of variation for PAP score expressed as a percent of the mean. ^2^ expressed in millimeters of mercury (mmHg), with a lower EPD being more favorable.

## Data Availability

Data are owned by Sitz Angus Ranch. Access to data can only be obtained by permission from the Sitz Angus Ranch.

## Abbreviations

The following abbreviations are used in this manuscript:

PAP	Pulmonary arterial pressure
HAD	High altitude disease
HMD	High Mountain Disease
RHF	Right-sided heart failure
mmHg	Millimeters of mercury
AAA	American Angus Association
AICc	Akaike’s Information Criterion adjusted for small sample sizes
EPD	Expected Progeny Differences
CV	Coefficient of variation
